# Evaluation of microRNA-10a and microRNA-210 as Biomarkers in Sepsis Patients With Acute Kidney Injury

**DOI:** 10.1155/ijne/1555811

**Published:** 2024-12-12

**Authors:** Hani Susianti, Catur Suci Sutrisnani, I. P. Adi Santosa, Wahyu Febrianto, Amanda Yuanita Kusdjianto, Kevin Putro Kuwoyo, Elita Riyu

**Affiliations:** ^1^Clinical Pathology Department, Faculty of Medicine, Universitas Brawijaya, Malang, Indonesia; ^2^Central Laboratory Department, RSUD Dr. Saiful Anwar, Malang, Indonesia

**Keywords:** acute kidney injury, microRNA-10a, microRNA-210, sepsis

## Abstract

**Background:** Sepsis-associated acute kidney injury (AKI) is a condition that increases in-hospital mortality and the risk of progression to CKD. The current method of detecting AKI, which relies on increased serum creatinine levels or a decrease in urine output, has low sensitivity. Early diagnosis and appropriate intervention in AKI can lead to improved patient outcomes. Several low molecular weight proteins and microRNAs detected in AKI are considered early biomarkers of AKI, such as miR-10a-5p and miR-210-3p.

**Method:** A cross-sectional study was conducted among 62 participants, consisting of 26 sepsis patients with AKI, 26 sepsis patients without AKI, and 10 healthy controls. AKI was determined according to KDIGO criteria. MicroRNA expression was analyzed using reverse transcription quantitative polymerase chain reaction (RT-qPCR). Statistical analysis was obtained using the Kruskal–Wallis test, Spearman's correlation coefficient, and ROC curve analysis.

**Result:** The median miR-10a-5p expression of the healthy controls versus sepsis with AKI versus sepsis without AKI groups was 10.38 (5.50–33.82) versus 10.32 (3.32–31.53) versus 9.76 (0.32–97.36), while the median miR-210-3p expression was 0.20 (0.03–0.41) versus 0.38 (0.04–1.24) versus 0.29 (0.06–1.67), respectively, with *p* = 0.721 for miR-10a-5p and *p* = 0.013 for miR-210-3 p. A significant increase in miR-210-3p expression was found in the sepsis with AKI compared to the healthy controls (*p* = 0.013) and sepsis without AKI (*p* = 0.034). miR-210-3p significantly correlated with creatinine and urea serum level (*p* < 0.05); miR-10a-5p did not have a significant correlation. The sensitivity and specificity of miR-10a-5p were 61.5% and 47.2%, and miR-210-3p were 84.6% and 63.9% for determining AKI.

**Conclusion:** The study's findings revealed a significant increase in miR-210-3p expression in sepsis patients with AKI, indicating its potential as a promising biomarker for determining AKI. This discovery demonstrates that the diagnostic performance of miR-210-3p surpasses that of miR-10a-5p, providing a more accurate biomarker for diagnosing AKI in sepsis patients.

## 1. Introduction

Acute kidney injury (AKI) is a sudden abrupt kidney function marked by increased creatinine or decreased urine output over hours to days. It can be caused by prolonged renal ischemia, sepsis, and nephrotoxins. Approximately, 1% of hospital admissions present with AKI upon admission in the United States of America. While hospitalized, the incidence rate of AKI ranges from 2% to 5%, affecting up to 67% of patients admitted to the intensive care unit (ICU). The mortality rate for AKI in hospital is 40%–50%, and more than 50% in the ICU [1].

Sepsis results from an uncontrolled inflammatory reaction to infection and poses a serious threat to life. It is characterized by dysfunction in multiple organs, and the kidney is one of the earliest organs to be injured during sepsis. In septic shock, approximately two-thirds of patients develop AKI. The development of AKI during sepsis is associated with increased patient morbidity, higher mortality rates, and significant effects on various organ functions [2, 3].

The principle of AKI management is to establish a diagnosis as early as possible because AKI requires supportive therapy to prevent further damage according to the cause, ranging from fluid management and antibiotics in sepsis to renal replacement therapy. Current biomarkers, such as creatinine used in the KDIGO definition of AKI, do not reflect real-time conditions due to their long half-life [4]. Thus, several low molecular weight proteins and microRNAs detected in AKI patients' serum are considered promising biomarkers of AKI [5]. The pathway that contributes to developing AKI in sepsis involves several signal pathways such as CJun kinase enzyme (JNK), nuclear factor-*κ*B (NF-*κ*B), phosphatase, and tension homolog (PTEN). MicroRNAs play a role in this mechanism [6]. Some promising biomarkers microRNAs include microRNA-10a-5p (miR-10a-5p) and microRNA-210-3p (miR-210-3p) [7].

MiR-10a-5p is a renal tubule-specific miRNA released from the kidney tissue upon injury. MiR-10a-5p plays a role in regulating the inflammatory response in the kidney. MiR-10a-5p may serve as a mediator in controlling renal inflammation. In mouse models of ischemia injury, renal reperfusion, and streptozotocin-induced diabetic nephropathy, miR-10a-5p expression was found to be decreased. In addition, some studies identify urine miR-10a-5p as a sensitive and specific biomarker for kidney injury. However, further research is needed to understand the role of miR-10a-5p in AKI, as there are still very few studies on this subject [8].

MiR-210-3p also has a vital role in AKI cases. Several studies have shown that miR-210-3p can improve angiogenesis and perfusion recovery in cases of ischemia, which causes AKI. Research conducted in mice showed that miR-210 regulates angiogenesis through the vascular endothelial growth factor (VEGF) signaling pathway after kidney ischemia/reperfusion injury [9]. In addition, other studies have shown that miR-210 can suppress the production of reactive oxygen species (ROS), which exacerbates kidney damage in AKI [10]. However, further research is needed to understand the role of miR-210 in AKI cases, especially as a biomarker, because there are few studies on this subject [9].

This study investigates the expression of miR-10a-5p and miR-210-3p in sepsis patients with kidney dysfunction (AKI) in the Indonesian population, which has not been previously investigated. This study distinguishes itself from prior research by comparing miRNA expression in two sepsis groups: with and without kidney dysfunction, in addition to healthy controls.

## 2. Methods

### 2.1. Participants' Samples

This cross-sectional study delves into the differences in miR-10a-5p and miR-210-3p expressions in healthy controls, sepsis patients with AKI, and sepsis patients without AKI. miR-10a-5p and miR-210-3p expressions were analyzed by reverse transcription quantitative polymerase chain reaction (qRTPCR). This study, conducted in the inpatient ward at Dr Saiful Anwar Hospital Malang and RSSA Central Laboratory from October 2023 until March 2024, could provide crucial insights into understanding sepsis and AKI. The inclusion criteria were that the participants were 18–65 years old and did not suffer from chronic kidney disease or malignancy disease.

The participants in this study underwent a comprehensive laboratory examination, a standard procedure for sepsis patients. The AKI was determined according to the KDIGO criteria. The demographic and other data were thoroughly searched from medical records. The institutional ethics board of Dr Saiful Anwar General Hospital has approved the study (ethical number 400/122/K.3/102.7/2023). All participants were asked to sign a consent form (informed consent), further solidifying the study's adherence to ethical guidelines.

### 2.2. microRNA Isolation

The microRNA isolation process involved collecting and storing serum samples at −80 degrees C until the isolation with a microRNA isolation kit (Tiangen Biotech). In brief, we add 900 *μ*L buffer MZA to 200 *μ*L serum or plasma, vortex for 30s to mix thoroughly, and then invert up and down to mix. Then, it was incubated at room temperature for 5 min to separate nucleic acids and protein. Then, 200 *μ*L chloroform was added to the supernatant and shaken for 15 s, incubating at room temperature for 5 min then centrifuged for 15 min at 12,000 rpm at 4°C. After centrifugation, the sample is separated into three phases: an upper, colourless, aqueous phase containing RNA; a white interphase; and a lower yellow organic phase. Then, we transfer the aqueous phase to a new tube and proceed to the next step. Add 2× the volume of ethanol (96%–100%) to the aqueous phase solution and mix thoroughly by pipetting up and down several times, then transfer the obtained liquid and any precipitate that may have formed into an RNase-free column miRelute, incubate for 2 min, then centrifuge at 12,000 rpm for 30 s. Discard the flow-through after centrifugation, and keep the column miRelute. Add 700 *μ*L buffer MRD to the column miRelute, incubate for 2 min, then centrifuge for 30 s at 12,000 rpm (repeat two times this step). Centrifuge at 12,000 rpm for 2 min at room temperature and discard the 2 mL collection tube. Then, we transfer the column miRelute to a new 1.5 mL RNase-free centrifuge tube, add 15–30 *μ*L RNase-free ddH2O directly onto the miRelute column membrane to avoid RNase contamination and incubate 2 min at room temperature. Then, centrifuge for 2 min at 12,000 rpm to elute the RNA. The microcentrifuge tube containing the eluted microRNA was stored at −80°C until qRTPCR examination.

### 2.3. qRTPCR

In brief, make a cDNA mastermix according to the kit procedure (Tiangen Biotech). Set the PCR machine/incubator with the program: Step 1: Incubate at 42°C for 60 min, Step 2: Inactivate at 95°C for 5 min, Step 3: Cool at 4°C. Store the cDNA that has been synthesized at 4°C/freezer. The primer is specific for miR-10a-5p and miR-210-3p with the housekeeping genes being hsa-U6 [11]. RTPCR detection was conducted using SYBR Green along with specific commercially available probes (Tiangen Biotech) for each miRNA of interest. The preparation of the master mix and the temperature cycling were carried out according to the manufacturer's guidelines. Prepare all real-time regents (ddH2O, master mix, and LNA primer), vortex, spindown the reagent, and dilute cDNA 80x in ddH2O. Make a qPCR master mix in the qPCR tube, mix the qPCR reagent, and then spindown for 2 min. Perform qPCR amplification according to the protocol. The results were analyzed using the Delta Delta CT(2-ΔΔCt) method to obtain the relative ratio of miR-10a-5p and miR-210-3p expression with housekeeping genes such as U6.

### 2.4. Statistical Analysis

The descriptive analysis described the median, minimum, and maximum miR-10a-5p and miR-210-3p expression levels between study groups. The Mann–Whitney test was performed to assess the comparison between the two study groups, and the Kruskal–Wallis test was performed to evaluate the comparison of miRNA expression between three study groups with a significance value of < 0.05 using SPSS Version 23 software. The correlation between miR-210-3p and miR-10a-5p with creatinine and urea serum levels was analyzed using the Pearson test or Spearman nonparametric statistics. Diagnostic tests were performed using the ROC curve, sensitivity, and specificity.

## 3. Results

### 3.1. Characteristics of Study Participants

The mean age of the participants was 60.08 ± 13.34 for sepsis patients with AKI and 57.88 ± 18.14 for sepsis patients without AKI. The hemoglobin levels were significantly lower in patients with sepsis than in healthy controls; however, there was only a small, insignificant difference in hemoglobin levels between patients with and without AKI. The levels of AST, ALT, and total bilirubin seem higher in sepsis patients than in healthy controls, while creatinine and albumin seem lower in sepsis patients than in healthy controls (Table 1).

### 3.2. microRNA-10a and microRNA-210 Expression

The median miR-10a-5p expression in healthy controls versus sepsis without AKI versus sepsis with AKI groups was 10.38 (5.50–33.82) versus 9.76 (0.32–97.36) versus 10.32 (3.32–31.53), while the median hsa-miR-210-3p expression was 0.20 (0.03–0.41) versus 0.29 (0.06–1.67) versus 0.38 (0.04–1.24), respectively (Table 2).

**Figure 1:**
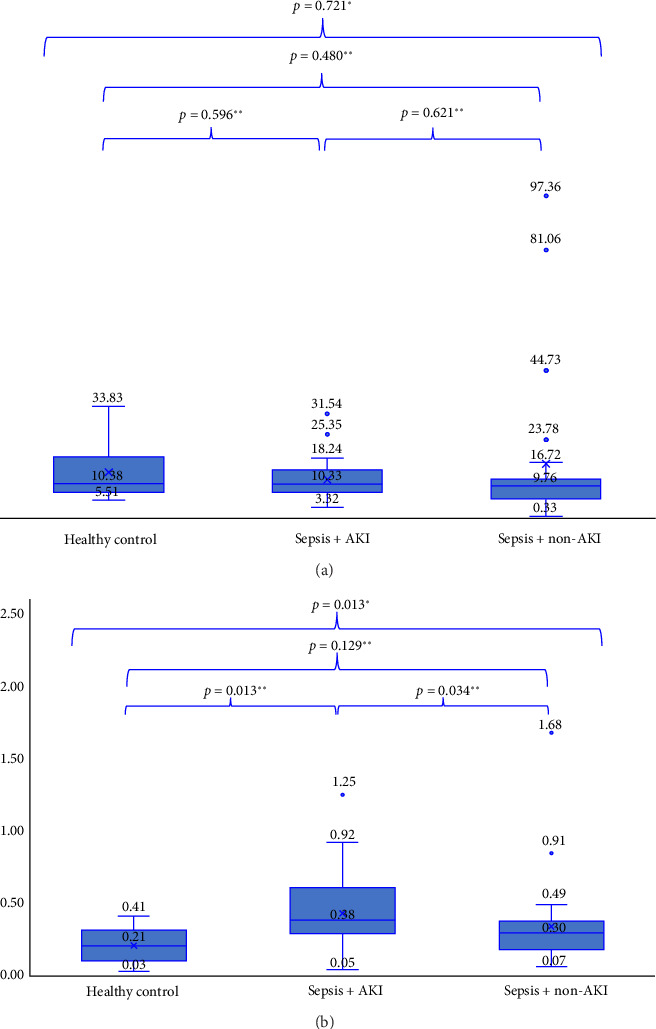
Boxplot expression of miR-10a-5p (a) and miR-210-3p (b) (Note: ∗Kruskal–Wallis; ∗∗Mann–Whitney).

Our analysis revealed a statistically significant difference (*p* = 0.013) in miR-210-3p expression between the three groups: healthy controls, sepsis patients with AKI, and sepsis patients without AKI (Figure 1(b)). This indicates that the expression of miR-210-3p is significantly different between the groups. Further multiple comparisons showed that the expression of miR-210-3p was significantly increased in the sepsis patients with AKI compared to the healthy controls group (*p* = 0.013) and sepsis without AKI (*p* = 0.034). In contrast to miR-210-3p, the expression of miR-10a-5p did not differ significantly (*p* > 0.05) between the three groups (healthy controls, sepsis with AKI, and sepsis without AKI), as shown in Figure 1(a). This finding suggests that miR-10a-5p may not be a useful indicator for identifying AKI in patients with sepsis.

### 3.3. Correlation Between miR-10a-5p and miR-210-3p With Kidney Function Tests

An analysis of kidney function tests, including creatinine and urea serum levels (Table 3), revealed a significant correlation between miR-210-3p expression and these markers (*p* < 0.05). This suggests that miR-210-3p may serve as a potential biomarker for AKI in sepsis patients. In contrast, miR-10a-5p expression did not significantly correlate with kidney function tests, indicating its limited usefulness in this context.

### 3.4. AUC, Sensitivity, and Specificity of miR-10a-5p and miR-210-3p

The analysis of receiver operating characteristic (ROC) curves (Table 4) revealed that miR-210-3p displayed a higher sensitivity (84.6%) and specificity (63.9%) for diagnosing AKI compared to miR-10a-5p (sensitivity: 61.5%; specificity: 47.2%). The 95% confidence intervals (CI) for miR-10a-5p and miR-210-3p were 0.350–0.640 and 0.652–0.902, respectively.

## 4. Discussion

Our study evaluated the expression of miR-10a-5p and miR-210-3p in 62 samples, consisting of 10 healthy controls, 26 sepsis patients without AKI, and 26 sepsis patients with AKI. Septic AKI has a poor prognosis; the reliance on serum creatinine and urine output as diagnostic indices for AKI has several limitations. microRNAs regulate inflammation, which plays a vital role in the pathogenesis of AKI, and several studies have used microRNA to diagnose AKI patients [12]. microRNAs are linked to the pathogenesis of septic AKI and are the most encouraging biomarkers in transcriptomics [6]. Our study showed that most subjects with sepsis with AKI were male gender. This result differed from Lin's study, which reported on sepsis patients with AKI; the number of male and female samples was almost the same [13].

The expression of miR-210-3p increased in this study; this is similar to the research conducted by Wang et al. and Fan et al., where there is an increase in miR-210 expression in AKI patients, mainly induced by sepsis [6, 10]. miR-210-3p works by being induced by hypoxia-inducible factor 1-alpha (HIF1-*α*). In cells experiencing hypoxia followed by reduced blood flow, HIF is stimulated to take control over the endothelium. HIF1-*α* increases the expression of miR-210, leading to enhanced glycolysis as well as endothelial proliferation and differentiation. Ultimately, this results in angiogenesis and increased blood flow. The angiogenesis mediated by miR-210-3p is also facilitated by Ephrin ligands and their Eph receptors, which play a role in vascular remodeling. The interaction between EphA2 and Ephrin-A3 can regulate angiogenesis. miR-210 is capable of reducing the modulation of Ephrin-A3, thereby enhancing the signaling of VEGF, which in turn stimulates capillary-like formation and the chemotaxis of endothelial cells in response to VEGF [10, 14, 15]. In addition, miR-210-3p is a miRNA-mediated signaling pathway in sepsis with AKI via NF-*κ*B. In the previous study stated that miR210HG, which is upregulated in LPS-induced immortalized human proximal tubular epithelial cells (HKC-8), activates the NF-*κ*B pathway by phosphorylating I*κ*B*α*, facilitating the movement of p65 into the nucleus and mitigating inflammatory responses. In contrast, inhibiting the NF-*κ*B signaling pathway has shown to enhance renal function [6, 16]. According to Lorenzen et al., who studied 77 critically ill patients with AKI compared to 30 healthy controls, plasma miR-210-3p has shown to be a strong and independent predictor of survival. Moreover, miR-210-3p was demonstrated to be enriched in blood samples of AKI patients [17]. Lin et al. showed that miR-210-3p and miR-494 expression were significantly decreased in survivors with sepsis-induced AKI [13].

In our study, there was no significant difference in the expression of miR-10a-5p in the three groups. The study conducted by Wang et al. showed that the miR-10a in serum was not correlated with kidney injury [8]. Huo et al. showed that miR-10a-5p expression increased in patients with sepsis and AKI compared to sepsis patients without AKI [18]. In contrast, Zheng et al. showed that miR-10a-5p levels were significantly lower in sepsis patients compared to patients with infection and healthy [19]. Our study also showed that miR-10 expression was lower in non-AKI sepsis patients than in healthy controls and sepsis patients with AKI. Hence, there is an inconsistency about the results of miR-10a-5p expression in AKI with sepsis patient that need further study.

Our study revealed a significant positive correlation between miR-210-3p expression and both creatinine (*r* = 0.390, *p* < 0.05) and urea levels (*r* = 0.378, *p* < 0.05), suggesting a potential link to kidney function. These findings align with previous work by Lin et al., who identified miR-210 as one of the most upregulated miRNAs in a similar context. Notably, their study reported a stronger correlation between miR-210-3p and kidney function markers such as BUN (*r* = 0.9715, *p* < 0.05) and creatinine (*r* = 0.8869, *p* < 0.05) compared to our findings [12]. In our study, this significant correlation between miR-210-3p with creatinine and urea levels showed that when kidney damage increases, the expression of miR-210-3p also increases.

However, our study showed an insignificant correlation between the decrease in miR-10a-5p expression with creatinine and urea levels. Thus, the decrease in miR-10a-5p expression does not reflect the severity of kidney damage. The results of our study differ from the research of Wang et al., already revealing that mouse urine contains miR-10a and microRNA-30d (miR-30d) that are abundant in the kidneys. miR-10a-5p can help to protect the kidney by regulating proapoptotic protein Bim and the expression of inflammation signal by IL-12/IL-23p40. miR-10a-5p also highly expressed during nephrogenesis by nephron progenitors which reflect another mechanism of kidney protection. miR-10a-5p plays a protective role in the kidneys by modulating the proapoptotic protein Bim. Bim is part of the large Bcl-2 family, which consists of both proapoptotic and antiapoptotic proteins. An increase in the expression of the proapoptotic Bim protein, which leads to cell death in mutant kidneys, has been linked to a decrease in mmu-miR-10a expression in nephron progenitor cells. In addition, miR-10a helps safeguard the kidneys by regulating inflammation that is mediated by IL-12/IL-23p40 signals released from dendritic cells in response to TLR and TLR ligand interactions. The presence of IL-12/IL-23 is essential for the activation of Th1 and Th17 cells, which play a significant role in the inflammatory response. Consequently, miR-10a functions as a negative regulator of both innate and adaptive immune responses [8, 20, 21].

The statistical analysis shows a positive correlation between the severity of renal damage caused by reperfusion post–renal ischemia or streptozotocin-induced diabetes and the levels of miR-10a-5p and miR-30d in urine. Moreover, it also increased in patients with focal segmental glomerulosclerosis (FSGS) contrasted with the healthy group [8]. The difference in the results of our study and Wang's study is possibly due to differences in the type of sample and participants in the research. But as we explained before, there is inconsistency regarding the expression of miR-10a-5p in patients with AKI and sepsis, which needs further explanation for future studies.

In our study, the AUC, sensitivity, and specificity of miR-210-3p were 0.777, 84.6%, and 63.9%, respectively. Lin et al. also stated that the sensitivity of miR-210 was 81.0, the specificity was 80.9, and the AUC was 0.852 to diagnose AKI [13]. More than 4000 miRNAs have been found in humans, with approximately one-third of them playing significant roles in the human genome and cellular functions [22]. miRNAs represent the most potential biomarkers at the transcriptomic level due to their plentifulness and durability in bodily fluids, circulating miRNAs may act as disease signatures and new molecular biomarkers [23, 24]. The current research has emphasized the promise of miRNAs as biomarkers for septic AKI [6].

The results of our study indicated that the AUC, sensitivity, and specificity of miR-10a-5p were 0.495, 61.5%, and 47.2%, respectively. Our study's diagnostic performance of miR-10a-5p cannot be directly compared with other studies, as Huo's study focused on miR-10a for predicting 28-day mortality in septic patients with AKI, not determining the AKI diagnosis [18]. The AUC, sensitivity, and specificity of miR-10a or determining the 28-day survival rate of septic AKI were 0.75, 85.71%, and 67.92%, which provide a promising direction for future studies. The combined miR-29a and miR-10a-5p demonstrated a superior AUC (0.87) than the individual detection of miR-29a, miR-10a-5p, cystatin-C, creatinine serum, and kidney injury molecule (KIM-1). Tapparo's study further supports the role of miR-10a-5p in sepsis patients with AKI and its potential as a therapeutic target in AKI [11].

Our study's limitation was the small number of samples. We did not include the participants' therapy and comorbidities as variables in the analysis. We suggest performing next-generation sequencing (NGS) in the next study to identify specific genetic features, including mutations, variations, and other genomic characteristics of sepsis-associated AKI patients with high resolution and accuracy.

## 5. Conclusion

Sepsis, a life-threatening condition, can lead to morbidity, higher mortality rates, and significant organ dysfunction. The kidney, being one of the earliest organs to be affected, necessitates the early detection of AKI. Our study revealed a significant increase in miR-210-3p expression in sepsis patients with AKI. This finding underscores the potential of miR-210-3p as a promising biomarker for sepsis-induced AKI, surpassing the diagnostic performance of miR-10a-5p.

## Figures and Tables

**Table 1:** Characteristics data of study participants.

Parameters	Normal (*n* = 10)	Sepsis without acute kidney injury (*n* = 26)	Sepsis with acute kidney injury (*n* = 26)	*p* value
Age	32.00 ± 6.96^a^	57.88 ± 18.14^b^	60.08 ± 13.34^b^	0.001∗
Gender (*n*, %)				0.012*∗*^@^
Female	8 (80%)	18 (69.23%)	9 (34.62%)	
Male	2 (20%)	8 (30.77%)	17 (65.38%)	
Leukocyte (×10^3^/mm^3^)	8.23 ± 1.20^a^	16.88 ± 13.56^b^	20.92 ± 10.93^c^	0.001∗
Neutrophil-to-lymphocyte ratio (%)	2.34 ± 0.83^a^	13.80 ± 14.98^b^	15.66 ± 11.05^b^	0.001∗
Hemoglobin (g/dL)	13.54 ± 1.43^a^	10.46 ± 2.55^b^	10.39 ± 1.97^b^	0.020∗
Hematocrit (%)	41.00 ± 4.25^a^	32.02 ± 8.43^b^	30.90 ± 6.34^b^	0.010∗
Thrombocyte (×10^3^/mm^3^)	310.70 ± 52.49	242.19 ± 157.78	226.15 ± 105.17	0.196
Natrium (mmol/L)	138.30 ± 10.22	135.54 ± 10.23	136.92 ± 9.75	0.721
Potassium (mmol/L)	4.05 ± 0.38	4.07 ± 1.18	4.29 ± 1.07	0.692
Chloride (mmol/L)	103.00 ± 7.13	102.50 ± 8.92	104.04 ± 11.09	0.846
Urea (mg/dL)	17.56 ± 7.21^a^	75.94 ± 55.22^b^	160.07 ± 95.35^c^	0.001∗
Creatinine (mg/dL)	0.75 ± 0.11^a^	1.85 ± 1.82^a^	3.93 ± 3.28^b^	0.001∗
AST (U/L)	24.90 ± 19.88^a^	129.73 ± 270.75^b^	113.92 ± 160.85^c^	0.030∗
ALT (U/L)	26.60 ± 31.11	160.46 ± 614.33	53.08 ± 64.82	0.536
Total bilirubin (mg/dL)	0.20 ± 0.07^a^	2.05 ± 2.59^b^	2.81 ± 6.86^c^	0.001∗
Albumin (mg/dL)	4.20 ± 0.35^a^	2.50 ± 0.73^b^	2.68 ± 0.58^b^	0.001∗
Random blood sugar (mg/dL)	97.60 ± 25.52^a^	137.38 ± 62.29^b^	164.65 ± 80.48^c^	0.040∗

*Note:* Characteristics data are shown in mean ± SD, unless otherwise noted. All tests are done in Kruskal–Wallis test with Mann–Whitney post hoc, except denoted with @, were tested with Chi-square. Values followed by the same letter are not significantly different according to the statistical test. Asterisks (∗) denote significance.

*p* < 0.05.

**Table 2:** Expression of microRNA-10a-5p and microRNA-210-3p.

Parameters	Healthy controls (*n* = 10)	Sepsis without acute kidney injury (*n* = 26)	Sepsis with acute kidney injury (*n* = 26)
miR-10a-5p expression (median, min–max)	10.38(5.50–33.82)	9.76(0.32–97.36)	10.32(3.32–31.53)
miR-210-3p expression (median, min–max)	0.20(0.033–0.41)	0.29(0.06–1.67)	0.38(0.04–1.24)

**Table 3:** Correlation between miR-10a-5p and miR-210-3p with kidney function tests.

Parameters	Creatinine serum		Urea serum	
	*p*	*r*	*p*	*r*
miR-10a-5p expression	0.590	−0.070	0.723	−0.046
miR-210-3p expression	0.002	0.390	0.002	0.378

**Table 4:** AUC, sensitivity, and specificity of miR-10a-5p and miR-210-3p.

Parameters	AUC	Cutoff	Sensitivity (%)	Specificity (%)
miR-10a-5p expression	0.495 (*p *=* *0.943)	9.437	61.5	47.2
miR-210-3p expression	0.777 (*p *=* *0.001)	0.297	84.6	63.9

Abbreviation: AUC, area under curve.

## Data Availability

The data that support the findings of this study are available from the corresponding author on request. The data are not publicly available due to privacy or ethical restrictions.
